# Acupuncture treatment on idiopathic trigeminal neuralgia: A systematic review protocol

**DOI:** 10.1097/MD.0000000000014239

**Published:** 2019-01-25

**Authors:** Hao Liu, Xin-Wei Li, Jia Du

**Affiliations:** a Department of Acupuncture and Moxibustion, Tongde Hospital of Zhejiang Province; b Zhejiang Academy of Traditional Chinese Medicine, Hangzhou, Zhejiang Province, China.

**Keywords:** acupuncture, meta-analyses, protocol, randomized controlled trials, trigeminal neuralgia

## Abstract

**Introduction:**

The objective of this systematic review protocol is to provide the methods for evaluating the effectiveness and safety of acupuncture on the treatment of idiopathic trigeminal neuralgia (ITN).

**Methods and analysis:**

We will search randomized controlled trials (RCTs) on this subject in 8 electronic databases and they are Cochrane Central Register of Controlled Trials (CENTRAL), MEDLINE, Embase, Chinese Biomedical Literature Database (CBM), China National Knowledge Infrastructure (CNKI), the Wan-Fang Database, and Chinese Scientific Journal Database (VIP database). Other relevant literatures will be manually searched as a complement. Only RCTs related to acupuncture for ITN in English or Chinese will be included, without limitation of publication types. The risk of bias and trial quality will be assessed by the Cochrane collaboration's tool. All data will be analyzed by RevMan V.5.3.3 statistical software.

**Ethics and dissemination:**

Ethical approval is not necessary as this paper does not involve patient data. This protocol will be disseminated by a peer-review journal or conference presentation.

**Trial registration number:**

PROSPERO CRD42015022173

**Strengths and limitations of this study:**

This systematic review will evaluate the effectivity and safety of acupuncture treatment on idiopathic trigeminal neuralgia. Two authors will perform independently study selection, data extraction and quality assessment, in order to ensure that all included studies are not personal bias. The result of this systematic review may give clinicians more ways to assist patient in relieving trigeminal neuralgia.

This shortage of systematic review is due to language barriers, only 2 languages of the trials can be included, other related studies may be missing. Different methods of acupuncture and quality of methodologies may result in essential heterogeneity.

## 1 Introduction

Trigeminal neuralgia (TN), also known as prosopalgia or tic douloureux, is one of the most common craninal neuralgia with a neuropathic pain affecting the facial area limited to the distribution of one or more divisions of the trigeminal nerve.^[1]^ The International Association for the Study of Pain (IASP) defined TN as “a sudden, usually unilateral, severe, brief, stabbing and recurrent episode of pain in the distribution of one or more branches of the fifth cranial nerve.”^[2]^ The annual incidence of TN is approximately 4.3 per 100,000 population^[3]^ with a female:male ratio of 1.7:1.^[4]^ In the latest classification of the International Headache Society,^[2]^ a distinction was made between classical and symptomatic TN: Classical TN (CTN) includes all cases without established etiology, i.e. idiopathic, as well as those with potential vascular compression of the fifth cranial nerve, whereas the diagnosis of Symptomatic TN (STN) is made in cases secondary to tumor, MS, structural abnormalities of the skull base, and the like. The pain associated with TN can often be of excruciating severity. The attacks are usually precipitated by non-noxious stimuli or common activities which stimulate trigger zones which is within the area of trigeminal nerve's distribution. Common antecedent stimuli include chewing, swallowing, brushing teeth, light touching, shaving, and washing. Anticonvulsive drugs, especially carbamazepine, have been shown to be effective in treating TN^[5]^ and are recommended as first-line therapy. When medical management fails to relieve the pain, the patients are always suggested to seek a surgeon. Though effective, there are limitation and shortage in pharmacological and surgical therapies on the treatment of TN. Pharmacologically, take carbamazepine and oxcarbazepine as examples, they have adverse effects including sedation, dizziness, ataxia, nausea, retching, and vomiting, and their therapeatic effect decreases with long-term use.^[6]^ Surgical procedures currently include glycerol rhizotomy (GR),^[7]^ partial sensory rhizotomy (PSR),^[8]^ radiofrequency thermorhizotomy (RF-TR),^[9]^ percutaneous ballon microcompression (PBC),^[10]^ stereotactic radiosurgery (SRS),^[11]^ and microvascular decompression (MVD).^[8]^ These methods all purposely damage the trigeminal nerve, trigeminal ganglion, or trigeminal root in controlled manner. Other procedures are intended for alleviating trigeminal neuralgia by relieving compression of the nerve at some point along its course. However, MVD may be more suitable because of the lowest rate of pain recurrence.^[12]^

Acupuncture is an effective method to improve trigeminal neuralgia and its adverse effect is almost none. Acupuncture, as a part of the traditional Chinese medicine, has been used for 3000 years and it is generally regarded as a safe and effective measure to relieve pain.^[13]^ Either manual acupuncture or electroacupuncture is normally applied to stimulate pain points of the body to unblocking the flow of qi for alleviating pain. Physiological researches reported that acupuncture stimulates the changes in the nervous system, particularly in pain inhibitory pathways.^[14]^ However, the mechanisms of acupuncture to successfully control or treat trigeminal neuralgia remain unclear in pathophysiology.

## 2 Objectives

This systematic review aims to evaluate the evidence for the effectiveness and safety of acupuncture treatment of ITN.

## 3 Methods and analysis

### 3.1 Study registration

This systematic review protocol has been registered on PROSPERO 2015 (registration number: CRD42015022173). Our review will develop following the Preferred Reporting Items for Systematic Reviews and Meta-Analyses (PRISMA) statement guidelines.

### 3.2 Types of studies

Only randomized controlled trials (RCTs) in English or Chinese are eligible for inclusion without restriction of publication type. Incomplete RCTs such as quasi-RCTs and randomized crossover designs will be excluded. Blinding will not be prioritized with the first sift-prescreening.

### 3.3 Participants

Eligible participants were those who were diagnosed with idiopathic trigeminal neuralgia based on international classification of headache disorders, 3rd edition of Headache Classification Committee of the International Headache Society. MRI must be employed to exclude secondary causes. There will be no restrictions on gender, intensity, frequency, and duration of ITN. But the age will be required to be older than 18.

### 3.4 Intervention

All types of acupuncture intervention in the treatment group will be eligible: either manaully or electrically, including fire needling, 3-edged needling, auricular acupuncture and pyonex. Eligible control group will have to be no treatment, sham acupuncture, placebo, and Western drug.

Studies with the following comparisons will be included:

1. Acupuncture versus drug therapy.
2. Acupuncture combined with other therapy versus the other therapy.
3. Acupuncture versus sham acupuncture.

Studies with the following comparisons will be excluded:

1. Comparison between different types of acupuncture.
2. Acupuncture combined with other therapy versus the other therapy plus Western drug or plus other treatment.

### 3.5 Outcome measures

#### 3.5.1 Primary outcomes

*Pain intensity*: relevant confirmed pain measurement scales such as visual analog scale (VAS), verbal rating scale (VRS), short-form McGill Pain Questionnaire (SF-MPQ)^[15]^, Numerical Rating Scale (NRS) will be analysed.

#### 3.5.2 Secondary outcomes

1. *Quality of life*: Any available assessments, such as EuroQol Survey (EQ-5D), and American Chronic Pain Association ranging from 0 (nonfunctioning) to 10 (normal daily activities)
2. Recurrence rate
3. Adverse effects (relevant symptoms caused by acupuncture)

### 3.6 Search methods for identification of the studies

#### 3.6.1 Electronic searches

According to the search strategy of Cochrane handbook guidelines,^[16]^ we will search the following electronic databases from inception to August 2018:

1. Cochrane Central Register of Controlled Trials (CENTRAL)
2. MEDLINE
3. Embase
4. China National Knowledge Infrastructure (CNKI)
5. Chinese Biomedical Literature Database (CBM)
6. Chinese Scientific Journal Database (VIP)
7. Wanfang Database

Following key words will be used: trigeminal neuralgia, prosopalgia, acupuncture, electroacupuncture, fire needling, 3-edged needling, pyonex, acupoint, intradermal needling, auricular acupuncture, randomized controlled trial, controlled clinical trial, and multicenter study.

The full list of the search strategy for PubMed is shown in Table 1. Modified search strategy will be used for other electronic databases.

**Table 1:**
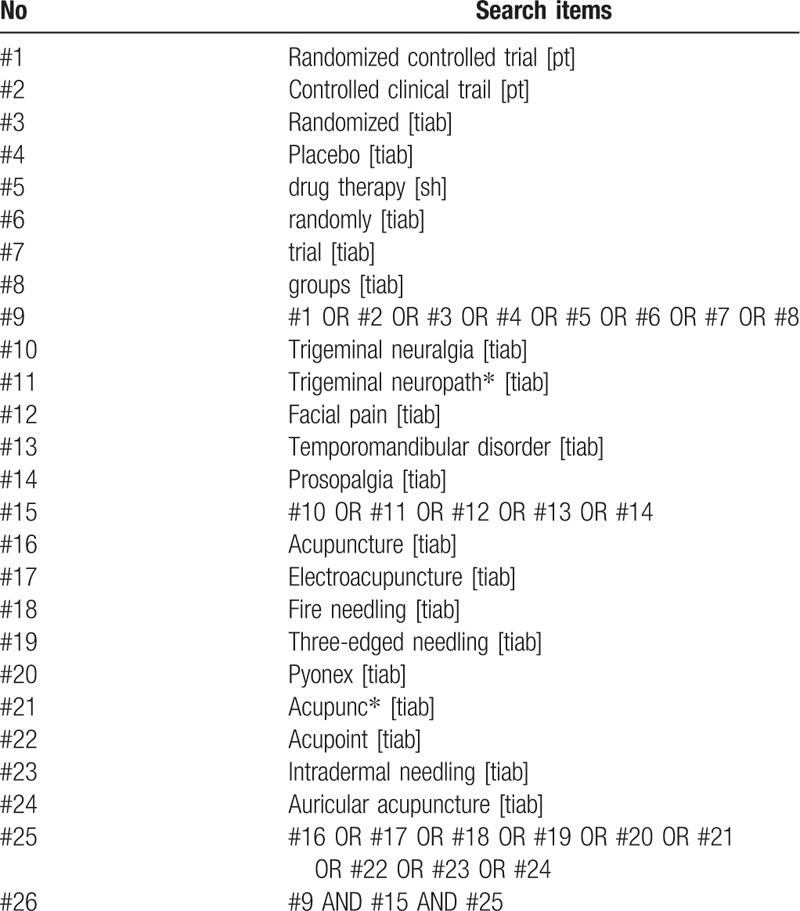
PubMed search strategy.

### 3.7 Searching other resources

Any related conference abstracts, unpublished or ongoing trails will also be included as supplementary.

### 3.8 Data collection and analysis

#### 3.8.1 Selection of studies

**Figure 1:**
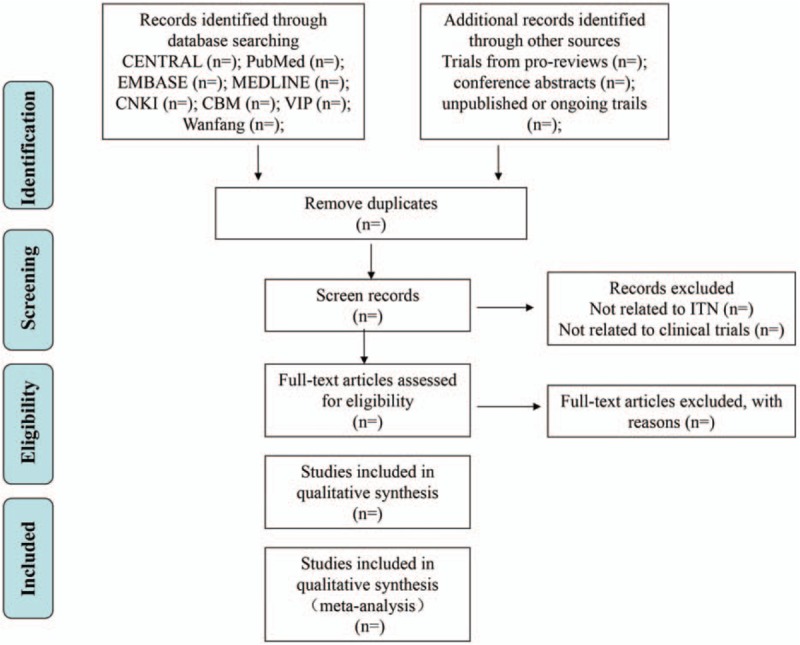
Flow diagram of selection process.

Previous training has enabled all reviewers to understand the methods of this review and use relevant management procedures. Two reviewers (HL and XWL) will independently screen the title and abstract of literatures according to inclusion and exclusion criteria; the full text will be reviewed when necessary. The articles that are not completely RCTs or duplicate ones will be removed. The third reviews (JD) will determine any disagreements by discussion. The detail of the selection procedure is presented in a Preferred Reporting Items for Systematic Reviews and Meta-Analyses (PRISMA) flow chart (Fig. 1)

#### 3.8.2 Data extraction and management

Two reviewers (HL and XWL) will conduct the data extraction with recognized data extraction form founded by all reviewers, including author, age, country, year of publication, characteristics of participants, intervention, randomized method, blinding, control treatment, main outcomes, and adverse events. If 2 reviews have any diverse comment, the argument will be discussed and determined by the third reviewers (JD). All electronic results will be uploaded to a database created by EndNote software (V.X6).

#### 3.8.3 Assessment of risk of bias in included studies

The risk of bias will be assessed in accordance with the Cochrane Collaboration Risk of Bias Tool.^[17]^ Two reviewers (HL and XWL) will independently assess methodological quality using the following 6 aspects: random sequence generation; allocation concealment; blinding; incomplete data; selective outcome reporting; other bias. Types of acupuncture, duration, and distribution of trigeminal neuralgia, and the age of participants may also cause bias. All included trials will be categorized into 3 levels of bias: low risk, high risk, and unclear. Any disagreements will be discussed and determined by a third reviewer (JD).

#### 3.8.4 Measurement of the treatment effect

For dichotomous data, we will use the relative risk (RR) with 95% confidence intervals (CIs) to analyze the effects of treatment. For continuous data, we will use the weighted mean difference (WMD) or the standard mean difference (SMD) with 95% CIs, the former will be used to measure the same outcome with the same scales, and the latter will be applied to measure the other types.

#### 3.8.5 Dealing with missing data

If the extractions are unclear or incomplete, we will try to contact the corresponding author or first author to obtain the data by e-mail or telephone. If the data cannot be obtained, the article will be excluded. An intention-to-treat analysis (ITT) will be introduced to reduce the risk of bias.

#### 3.8.6 Assessment of heterogeneity

It is important that *I*^*2*^ statistic and *χ*^*2*^ test will be used to assess the heterogeneity. We consider that if *I*^*2*^ value^[16]^ is more than 50% and *P* value is <.10, included articles is proved to have a significant heterogeneity. A range of 0% to 40% indicates unconspicuous heterogeneity; a range of 30% to 60% indicates temperate heterogeneity; a range of 50% to 90% indicates essential heterogeneity; a range of 75% to 100% indicates considerable heterogeneity. If heterogeneity is detected, we will perform subgroup analysis to inquire the potential causes.

#### 3.8.7 Assessment of reporting biases

We will create funnel plots to detect reporting bias, when more than 10 trials are included in this review. Egger's regression method will also be used to evaluate the publication bias.^[18]^

## 4 Data synthesis

All data of included studies will be analyzed by Review Manager (RevMan V.5.3.3) from the Cochrane Collaboration. According to a heterogeneity result obtained by *χ*^*2*^ test and *I*^*2*^ statistic, we will select a random effects model and a fixed effects model, the former is appropriate for the low heterogeneity (*P > *.10, *I*^*2*^<50%), and the latter will be adopted the result of high heterogeneity (*P*<0.10, *I*^*2*^>50%). However, we will not accept considerable heterogeneity (*I*^*2*^>75%) in meta-analysis which cannot be explicated by methodological and clinical variation.

### 4.1 Subgroup analysis

If included data are available, we will produce subgroup analysis to evaluate the heterogeneity with the following factors:

1. Type of acupuncture intervention
2. Type of control (e.g., placebo/sham, Western medicine)
3. Level and duration of ITN
4. Classification of ITN (e.g., distribution of trigeminal nerve, unilateral or bilateral)

### 4.2 Sensitivity analysis

Sensitivity analysis is an analytical method to confirm the stability and reliability of the systematic review results through elimination of lower quality trials and different model analysis. Pooled effect size will be recalibrated whether the results are robust.

## 5 Discussion

ITN is defined as the “the world's worst pain” by patients,^[19]^ which have strong impact on their quality of life and social activities. Meanwhile, patients are also troubled by the side effect of medicine, adaptability of operation, and economical burden. In China, acupuncture is the most common therapeutic option for diseases with pain. It is proved by clinical reports^[20,21]^ and relevant animal experiments^[22–24]^ that acupuncture reduces the symptom of neurodynia. A previous systematic review cannot insufficiently prove the effectiveness and safety of acupuncture on trigeminal neuralgia due to the low quality of research methodology and restriction of language. Therefore, a high quality systematic review is needed. We hope that this systematic review will provide the current clinical evidence on the effectiveness and safety of acupuncture treatment for idiopathic trigeminal neuralgia, and can provide more useful information to the doctor in clinical practice and better choice for patients. This research may have some shortages including different types of acupuncture, the restriction of language and missing studies which could cause essential heterogeneity.

## Author contributions

**Data curation:** Xin-Wei Li.

**Project administration:** Jia Du.

**Writing – original draft:** Hao Liu.

Jia Du orcid: 0000-0003-4966-4425.
